# Assessment of polymeric mucin–drug interactions

**DOI:** 10.1371/journal.pone.0306058

**Published:** 2024-06-27

**Authors:** Hisanao Kishimoto, Caroline Ridley, Katsuhisa Inoue, David J. Thornton

**Affiliations:** 1 Department of Biopharmaceutics, School of Pharmacy, Tokyo University of Pharmacy and Life Sciences, Hachioji, Tokyo, Japan; 2 Wellcome Centre for Cell-Matrix Research, School of Biological Sciences, Faculty of Biology, Medicine and Health, Manchester Academic Health Sciences Centre, The University of Manchester, Manchester, United Kingdom; : Keele University School of Life Sciences, UNITED KINGDOM

## Abstract

Mucosal-delivered drugs have to pass through the mucus layer before absorption through the epithelial cell membrane. Although there has been increasing interest in polymeric mucins, a major structural component of mucus, potentially acting as important physiological regulators of mucosal drug absorption, there are no reports that have systematically evaluated the interaction between mucins and drugs. In this study, we assessed the potential interaction between human polymeric mucins (MUC2, MUC5B, and MUC5AC) and various drugs with different chemical profiles by simple centrifugal method and fluorescence analysis. We found that paclitaxel, rifampicin, and theophylline likely induce the aggregation of MUC5B and/or MUC2. In addition, we showed that the binding affinity of drugs for polymeric mucins varied, not only between individual drugs but also among mucin subtypes. Furthermore, we demonstrated that deletion of MUC5AC and MUC5B in A549 cells increased the cytotoxic effects of cyclosporin A and paclitaxel, likely due to loss of mucin-drug interaction. In conclusion, our results indicate the necessity to determine the binding of drugs to mucins and their potential impact on the mucin network property.

## Introduction

Mucus is a viscoelastic hydrogel that covers several epithelial surfaces of the body such as the gastrointestinal, intranasal, and respiratory epithelia. Mucus forms a physiological barrier to protect against infections, foreign particles and mechanical damage, and is also involved in the regulation of drug absorption through various surface tissues [1, 2]. The major structural components of mucus barriers are gel-forming mucins such as MUC2, MUC5AC, and MUC5B, which form disulfide-bonded polymer-based networks that underpin mucus viscoelasticity and its ability to act as a biological molecular sieve (a range of pore sizes between 20 and 400 nm) [3, 4]. Moreover, the complex chemical structure of mucins, such as highly O-glycosylated hydrophilic central mucin domains and non-O-glycosylated cysteine-rich hydrophobic protein regions, can also act as a selective physicochemical barrier and provides binding sites (hydrophobic or electrostatic binding) [2, 5]. Recently, there has been considerable interest in the interactions between mucins and drug compounds in terms of whether the mucus barrier plays an important role in efficient mucosal drug absorption [6, 7].

Mucosal-delivered drugs have to pass through the mucus layer before absorption through the epithelial cell membrane and into the circulatory system. Thus, mucin-drug interactions may be a key factor involved in drug bioavailability [8, 9]. This interaction mechanism is mainly governed by physicochemical interactions, which would have two consequences: 1) A change mucin viscoelastic properties due to mucin aggregation and 2) a decrease in free drug concentration due to drug binding to mucin. However, the detailed molecular basis has yet to be clarified.

In a recent study, we demonstrated that lipophilic cyclic peptide drugs such as cyclosporin A have the ability to interact with human polymeric mucins and induce aggregation of MUC5B [10]. Although detailed interaction mechanisms have been mainly examined for MUC5B, the differences in drug interactions among the three mucin subtypes have not yet been explored. Furthermore, the chemical properties of drugs involved in interactions with mucins remain unclear, and the influence of the interactions on drug absorption and efficacy have not been investigated. Therefore, it is important to comprehensively evaluate the interaction of polymeric mucins with drugs with different chemical profiles to characterize their potential influence on drug absorption and efficacy.

In this study, we have investigated the interaction between three different human polymeric mucins (MUC2, MUC5B, and MUC5AC) and several drugs with different physicochemical properties (e.g. lipophilicity and physiological charge). We selected nine model drugs, known to cross epithelial membranes mainly by passive diffusion, to assess interaction with mucins [9–11]. We examined the effect of model drugs on mucin sedimentation and aggregation behavior. Moreover, we estimated the binding affinity of the drugs to mucins by using fluorescence spectral analysis. Lastly, we found that deletion of the main two polymeric mucins, MUC5AC and MUC5B, in A549 cells increased cytotoxic effects of cyclosporin A and paclitaxel likely due to loss of mucin-drug interaction.

## Materials and methods

### Materials

Daptomycin, polymyxin B, cyclosporin A, paclitaxel, rifampicin, griseofulvin, antipyrine, theophylline, and 5-fluorouracil were obtained from Tokyo Chemical Industry Co., Ltd. (Tokyo, Japan). Cesium chloride (CsCl) was purchased from Melford (Ipswich, UK). All other reagents were of analytical grade.

### Polymeric mucin purification

MUC2, MUC5AC, and MUC5B were purified from LS174T cells and knockdown A549 cells by two-step isopycnic cesium chloride (CsCl) density gradient centrifugation (starting density of 1.4 g/mL and then 1.5 g/mL), as previously described [10]. Mucin containing fractions were detected using mucin specific antibodies (MAN-2I: MUC2 [12], MAN-5ACI: MUC5AC [13], and EUMUC5B: MUC5B [14]) after slot blot onto nitrocellulose and pooled for further analyses [10].

### Assessment of aggregation of polymeric mucins by drugs

The mucin aggregation mediated by drugs was assessed using a simple centrifugation assay. The mucins (200 μg/mL in PBS at pH 7.4) were incubated at 37°C for 1 hour with 1 mM each drug (dissolved in 10% DMSO) [10]. Each sample was subsequently centrifuged at 13,000 rpm (12,492 x g) for 10 min to separate mucins in the supernatant from those in the pellet. The pellet sample was resolubilized in an equal volume of 6 M urea. All samples obtained were loaded onto the nitrocellulose membranes using a Minifold II 72 well slot blot apparatus with a water suction vacuum, and then the mucins were analyzed by immunodetection [10].

### Effect of drugs on MUC5B size distribution

The influence of drugs on mucin self-interaction was assessed by analyzing changes in MUC5B sedimentation behavior, as described previously [15]. The MUC5B (200 μg/mL in PBS at pH 7.4) was incubated at 37°C for 1 hour with 1 mM of each model drug. The sedimentation profile of the MUC5B was determined using rate-zonal centrifugation on sucrose density gradients (10–35% (w/v)).

### Assessment of drug-mucin interaction by measurement of fluorescence emission spectra

To assess the drug-binding to mucin, fluorescence emission spectra of mucins (MUC2, MUC5AC, and MUC5B) were measured by using a Horiba FluoroMax 4 spectrometer (HORIBA scientific, Jobin Yvon, USA), as described previously [10]. A constant concentration of all mucins (10 μg/mL in PBS at pH 7.4) were analyzed with increasing concentrations of model drugs (rifampicin; 0 to 10 μM, griseofulvin; 0 to 20 μM, daptomycin, polymyxin B, cyclosporin A, paclitaxel, antipyrine, theophylline, and 5-fluorouracil; 0 to 200 μM). Samples were left to equilibrate for 30 min at 37°C and then fluorescence emission spectra were measured. The obtained fluorescence data were analyzed by Lineweaver-Burk equation for the determination of dissociation constants (*K*_d_) [16].


$$\frac{1}{\left(F_{0}-F\right)}=\frac{K_{d}}{F_{0}}\cdot \frac{1}{\left[C\right]}+\frac{1}{F_{0}}$$

*F*_0_ is the fluorescence intensity of polymeric mucins alone, *F* is the fluorescence intensity at increasing drug concentration, *K*_d_ is the dissociation constant, [*C*] is the drug concentration in sample.

### Establishment of endogenous MUC5AC and MUC5B knock-out A549 cell line

The gRNAs for genome editing using the CRISPR-Cas9 system were designed based on CRISPR design tools (http://crispor.tefor.net/) according to the cDNA sequences of the following accession numbers: human MUC5AC (NM_001304359) and human MUC5B (NM_002458) were obtained from GenBank^™^. The primer sets are listed in S1 Table. Double-stranded complementary gRNA oligos were cloned into an EF1-T7-hspCas9-T2A-GFP-H1-gRNA linearized SmartNuclease vector (System Biosciences, Mountain View, CA). A549 cells were transfected with the two types of CRISPR-Cas9 gRNA vectors using Lipofectamine 3000 (Invitrogen Carlsbad, CA). To obtain stable knock-out cell lines, the transfected cells were isolated as single cell subclones and the production of MUC5AC and MUC5B in subclones was detected by western blot analysis using specific antibodies [MUC5AC (Abcam, catalog no. ab198294), MUC5B (Proteintech, catalog no. 28118-1-AP), β-actin (Santa Cruz Biotechnology, catalog no. sc-47778)]. Likewise, mock cells were generated using an empty vector.

### Cytotoxic assay

Cytotoxicity was assessed by cell counting kit-8 (CCK-8, Dojindo Laboratories Kumamoto, Japan). MUC5AC/5B-knock-out A549 cells, along with A549 controls, were incubated for 24 hours after seeding, then exposed to various concentrations of drugs in FBS free culture medium for 72 hours. All stock solutions of the drugs (prepared in 100% DMSO) were diluted into the FBS free culture medium at concentrations less than 0.5%. Each concentration-response curve obtained from cytotoxic assay was fitted to the concentration-response stimulation nonlinear regression model and IC_50_ values were obtained by GraphPad Prism v9.2 software (San Diego, CA) [11].

### Data analysis

Data are presented as mean ± s.e.m. Statistical analyses were performed using Student’s t-test or one-way ANOVA followed by Dunnett’s method; *P* < 0.05 was considered statistically significant. Statistical analyses were performed with GraphPad Prism v9.2.0.

## Results

### A simple aggregation assay to investigate potential interaction between polymeric mucins and drugs

**Fig 1:**
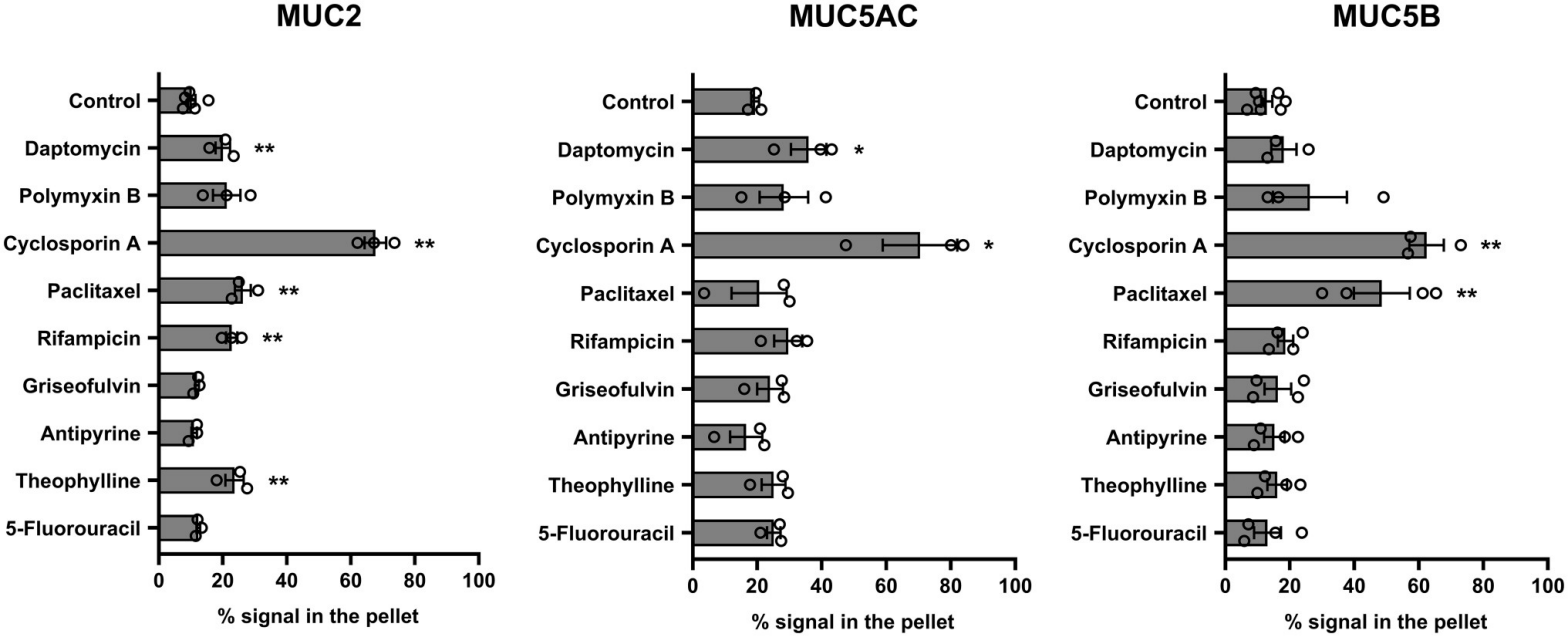
Effect of a range of drugs on aggregation of gel-forming mucins, MUC2, MUC5AC and MUC5B. Mucins were incubated with a range of drugs for 60 min at 37°C (daptomycin, polymyxin B, cyclosporin A, paclitaxel, rifampicin, griseofulvin, antipyrine, theophylline and 5-fluorouracil; each at 1mM) and subsequently centrifuged (10 min at 12,492 x g). Mucins in the supernatant and pellet were analyzed by immunodetection with the mucin-specific antibody probes, MAN-2I (MUC2), MAN-5ACI (MUC5AC), and EUMUC5B (MUC5B). Band intensities were quantified using the Odyssey Imaging system. Results are presented as the mean ± s.e.m. (n = 3–7) from 3 independent experiments. *P < 0.05, **P < 0.01 compared with control condition (one-way ANOVA followed by Dunnett’s method).

We have previously shown that the lipophilic cyclic peptides, cyclosporin A and daptomycin, interact with polymeric mucins (MUC2, MUC5AC, and MUC5B), and induce their aggregation [10]. Therefore, we first evaluated the potential interaction between these three polymeric mucins and several model drugs (daptomycin, polymyxin B, cyclosporin A, paclitaxel, rifampicin, griseofulvin, antipyrine, theophylline and 5-fluorouracil) using a simple centrifugation-based assay to detect mucin aggregation. As expected, cyclosporin A significantly increased aggregation of the three polymeric mucins, and daptomycin increased aggregation of MUC2 and MUC5AC (Fig 1). We found that paclitaxel, rifampicin, and theophylline aggregated MUC2, and paclitaxel also aggregated MUC5B. In contrast, no aggregation was induced with polymyxin B, rifampicin, griseofulvin, antipyrine, and 5-fluorouracil under the conditions of the assay.

### The effect of drugs on the sedimentation behavior of MUC5B

**Fig 2:**
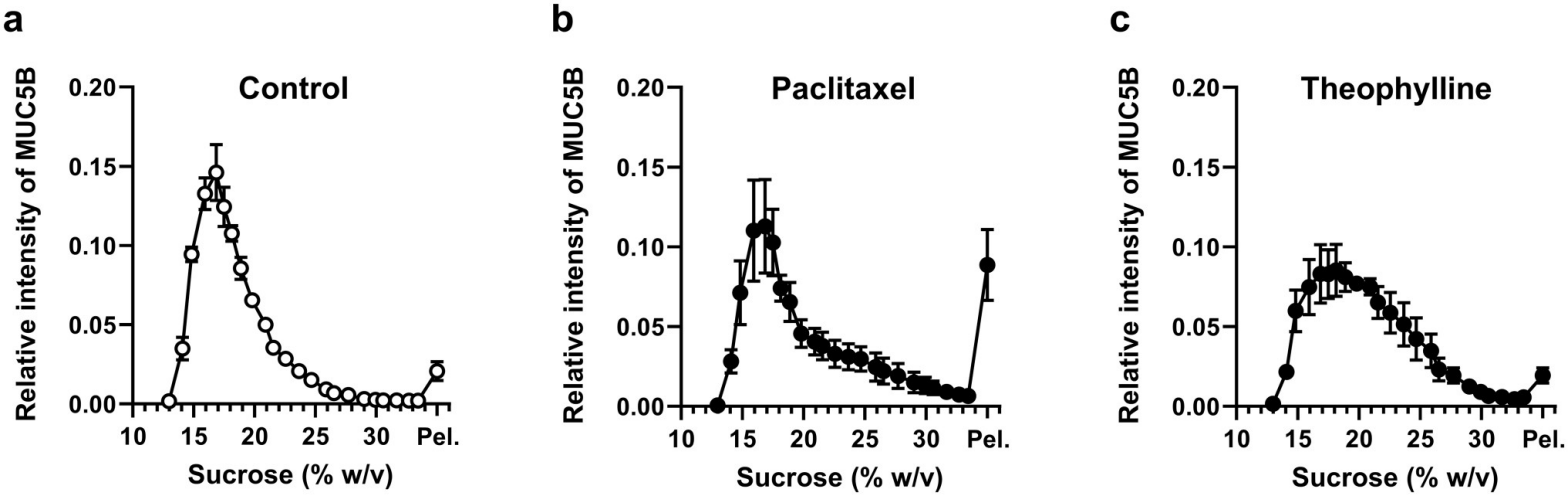
Sedimentation profiles of MUC5B in the presence of model drugs. Rate-zonal centrifugation of MUC5B (control; a), or with 1 mM paclitaxel (b), or 1 mM theophylline (c), performed in 10–35% (w/v) sucrose gradients in PBS. Mucins were detected in fractions by immunodetection using the MUC5B specific antibody (EUMUC5B) after slot blotting onto nitrocellulose, and the band intensities were quantified using the Odyssey Imaging system. The results are presented as the mean ± s.e.m. (n = 3–5) from at least 3 independent experiments. Pel., pellet sample.

We next explored in more detail whether drugs could influence the biophysical properties of polymeric mucins by assessing changes in the sedimentation behavior of MUC5B after incubation with each drug (Fig 2 and S1 Fig). We previously demonstrated that cyclic peptides such as cyclosporin A can induce aggregation of MUC5B and alter its sedimentation properties [10]. After rate-zonal centrifugation on 10–35% (w/v) sucrose gradients, in the absence of drugs, MUC5B was found mainly in the fractions containing 10–25% (w/v) sucrose, and only a small amount was pelleted (Fig 2a). After treated with 1 mM paclitaxel, the MUC5B sedimentation was altered and the proportion of MUC5B in the pellet fraction was significantly increased (Fig 2b). The percentage of MUC5B in the pellet was 8.9% increased from 2.1% without paclitaxel (Table 1). This result suggested that interaction with paclitaxel induced mucin aggregate formation, in agreement with the results presented in Fig 1. After incubation with 1 mM theophylline, MUC5B showed a broader sedimentation profile (Fig 2c and Table 1), which suggested that theophylline interacts with and causes self-association of MUC5B. However, it does not cause aggregation of MUC5B to the extent that it pelleted in the sucrose gradient, at least under the conditions studied. In contrast, the sedimentation profile of MUC5B was largely unaltered after incubation with 1 mM rifampicin, griseofulvin, antipyrine, and 5-fluorouracil (S1 Fig), suggesting that under the conditions of the experiment, any interaction with these compounds did not induce MUC5B self-association. Furthermore, we found that the results of a simple aggregation assay correlated with changes in sedimentation behavior of MUC5B in sucrose density gradients. Compared to untreated MUC5B, paclitaxel and theophylline induced MUC5B-aggregates were clearly observed in the sucrose gradients and in the simple sedimentation assay a higher proportion of MUC5B was found in the pelleted material. This suggests that the simple aggregation assay is a potentially useful method to assess drug-induced mucin aggregation.

**Table 1:** Percentage of MUC5B in sucrose gradient fractions.

Sucrose (% w/v)	Percentage of MUC5B in Rate-Zonal Fractions		
	Control	Paclitaxel	Theophylline
**10 ~ 15**	5.75 ± 1.77	2.87 ± 0.71	5.14 ± 2.72
**15 ~ 20**	73.54 ± 2.31	58.22 ± 10.01	51.55 ± 6.10**
**20 ~ 25**	15.00 ± 1.71	17.20 ± 4.07	29.25 ± 5.48*
**25 ~ 30**	2.76 ± 0.70	9.36 ± 3.61	9.73 ± 2.95*
**30 ~ 35**	0.88 ± 0.11	3.49 ± 1.13*	2.40 ± 0.16**
**Pellet**	2.07 ± 0.60	8.87 ± 2.22*	1.93 ± 0.48

Results are presented as the mean ± s.e.m. (n = 3–5) from 3 independent experiments. Results were compared with control conditions (one-way ANOVA followed by Dunnett’s method, *P < 0.05, **P < 0.01). The percentage of MUC5B in fractions across the sucrose gradient was calculated from Fig 2.

### Investigation of binding of various drugs to polymeric mucins by measuring fluorescence emission spectra

The measurement of changes in fluorescence spectra is a widely used and reliable method for assessing the interactions between proteins and drugs [17], and there are reports evaluating the interaction of drugs with bovine mucins [16]. To evaluate potential binding of each drug on gel-forming mucins, that did or did not result in promoting mucin self-association, we measured mucin fluorescence intensity after incubation with a range of concentrations of the drugs (S2–S4 Figs). For all drugs tested, fluorescence intensity decreased in a drug concentration-dependent manner, indicating binding to mucin. The calculated dissociation constants (*K*_d_) of the different drugs with mucins are shown in Table 2. The *K*_d_ values were found to vary between the drugs tested between different mucin subtypes and the mucin-drug interactions were classified into three groups: 1) Drugs that show similar interaction strength between mucin subtypes (griseofulvin, antipyrine, and 5-fluorouracil), 2) drugs that interact relatively more strongly with one or two mucins (cyclosporine A, daptomycin, rifampicin, and theophylline), and 3) drugs that show different interactions between mucin subtypes (polymyxin B and paclitaxel). It is noteworthy that the *K*_d_ values obtained did not correlate with the degree of mucin aggregation caused by these drugs or with the physicochemical properties of each drug (e.g. molecular weight or log *P* value).

**Table 2:** Dissociation constants (*K*_d_ values) of mucin.

	*MW*	*LogP*	*K*_*d*_ *(μM)*		
			*MUC2*	*MUC5AC*	*MUC5B*
**Daptomycin**	1620.69	-9.4	358.4 ± 59.2	123.2 ± 13.1	329.0 ± 84.3
**Polymyxin B**	1385.61	-7.2	106.4 ± 19.4	349.9 ± 98.9	1562.1 ± 280.9
**Cyclosporin A**	1202.64	3.64	157.0 ± 16.3	146.7 ± 62.2	847.4 ± 117.6
**Paclitaxel**	853.92	3.54	163.1 ± 39.1	87.6 ± 7.0	288.0 ± 24.9
**Rifampicin**	822.95	2.56	49.7 ± 6.2	15.0 ± 1.5	44.5 ± 7.1
**Griseofulvin**	352.77	2.17	45.3 ± 9.6	25.4 ± 1.6	29.9 ± 5.0
**Antipyrine**	188.23	0.38	393.1 ± 26.2	302.0 ± 6.7	386.0 ± 49.3
**Theophylline**	180.17	-0.02	390.2 ± 46.5	185.4 ± 5.6	188.9 ± 7.2
**5-Fluorouracil**	130.08	-0.66	135.6 ± 12.1	124.1 ± 1.5	142.8 ± 17.0

Results are presented as the mean ± s.e.m. (n = 3–6) from 3 independent experiments.

### Effects of mucin-drug interactions on their cytotoxic effect

**Fig 3:**
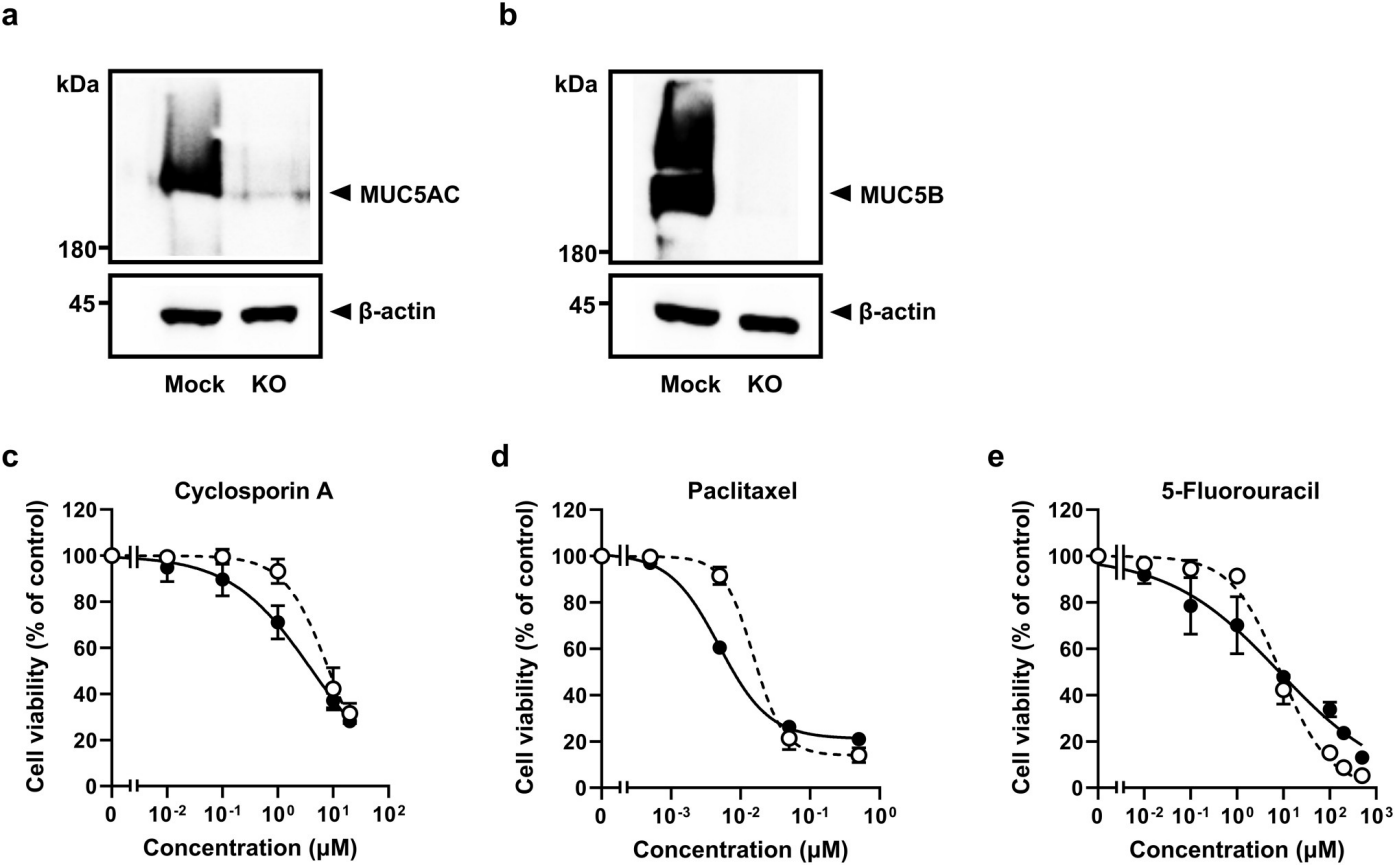
Alterations in drug-induced cytotoxicity in A549 cells by knock-out of MUC5AC and MUC5B. The expressions of (a) MUC5AC and (b) MUC5B in A549 cells were verified by western blotting. Cell viability was assessed by CCK-8 assay using mock cells (open circle) and MUC5AC/5B-KO (closed circle) treated with different concentrations of: (c) cyclosporin A (1 nM–20 μM); (d) paclitaxel (0.5 nM–5 μM); (e) 5-fluorouracil (10 nM–500 μM) for 72 hours. The dotted line represents the mock cell controls. Cell viability (measured as absorbance) is expressed as % untreated control. Results are presented as the mean ± S.D. from 3 independent experiments. The solid curves were drawn by fitting the data to the three-parameter logistic equation by GraphPad Prism v9.2. The blots were cropped and full-length blots are presented in S1 Raw images.

Our results have identified that drugs can bind to mucins, and some can induce mucin aggregation. To assess the potential pharmacological consequences for mucin-drug interaction, we established a gel-forming mucin (MUC5AC and MUC5B) knock-out A549 cell line (Fig 3a and 3b) and examined the impact of mucin-depletion on cell cytotoxicity. Mucin secretion from the mock cells was detectable 24 hours after seeding and continued to increase in a time-dependent manner for up to 72 hours. In contrast, no mucin was detected from the knock-out cells (S5 Fig). Treatment of mock and MUC5AC/MUC5B knock-out cells with 5-fluorouracil, cyclosporin A and paclitaxel, showed dose-dependent increased cytotoxicity, and IC_50_ values are presented in Fig 3c–3e and Table 3. Cell viability was decreased after treatment with cyclosporin A and paclitaxel to a greater extent in MUC5AC/MUC5B knock-out cells compared with the mock cells (Fig 3c and 3d). In the case of 5-fluorouracil, the dose-response pattern differed in the mucin-deficient cells, and cell survival decreased within the concentration range under 10 μM compared with that in mock cells (Fig 3e). Furthermore, the IC_50_ values for cyclosporin A and paclitaxel were significantly reduced by a third in knock-out cells compared to mock cells, while the IC_50_ value for 5-fluorouracil was unchanged (Table 3). These results suggest that absence of MUC5AC and MUC5B production increased the cytotoxic effects of these model drugs, likely due to loss of mucin-drug interaction and an increase in free drug concentration.

**Table 3:** IC_50_ values for various anticancer drugs.

	IC_50_	
	Mock cells	MUC5AC/5B KO cells
**Cyclosporin A**	13.73 ± 2.21 μM	4.26 ± 0.72 μM *
**Paclitaxel**	13.41 ± 0.12 nM	4.78 ± 0.19 nM **
**5-Fluorouracil**	8.86 ± 1.27 μM	9.39 ± 1.65 μM

The IC_50_ values were calculated from the concentration-response curves in the MUC5AC/5B knock-out, and mock cells treated with model drugs. Results are presented as the mean ± s.e.m. (n = 3) from 3 independent experiments.

*P < 0.05,

**P < 0.01 as compared to control condition (Student’s t-test).

## Discussion

Mucin-drug interactions are important when considering efficient drug absorption, however, there are no reports that have systematically evaluated the interaction of drugs with different chemical profiles and human polymeric mucins. In this study, we have demonstrated that the potential interaction between human mucins and various drugs by relatively simple and rapid centrifugal method using purified human polymeric mucins (MUC2, MUC5B, and MUC5AC). Furthermore, the resultant loss of mucin-drug interactions in our knock-out cell line has provided valuable insights into the potential influence of mucins on drug absorption and pharmacological effects. Our results thus underscore the need for comprehensive investigations of mucin-drug interactions, to define the detailed molecular basis of these interactions.

Although the detailed molecular mechanisms underlying the interactions between drugs and mucus components during epithelial diffusion remain unclear, our findings provide new insights for addressing this issue. In the present study, we demonstrated that paclitaxel, a neutral hydrophobic molecule, interacted with and likely induced the aggregation of MUC5B and MUC2, as shown by a simple centrifugal method and by sucrose density gradient centrifugation. In addition, our data demonstrated that the removal of mucin-drug interactions by knocking-out endogenous mucins resulted in changes in the cytotoxic effects of paclitaxel (and cyclosporin A). Under physiological conditions, the mucus forms a barrier against foreign particles by acting as a biological molecular sieve formed by mucin networks, as well as providing potential binding sites. However, almost all commercially available pharmaceutical compounds are smaller than globular proteins such as albumin (15–650 kDa), which can freely diffuse through the mucus layer [3, 18, 19]. This suggests these small molecule compounds are less likely to be limited by biomolecular sieve properties of the mucin network. Therefore, it is important to consider interactions with protein-rich regions of mucins and/or binding to mucin O-glycans as potential hurdles to diffusion of drugs through the mucus layer. In our previous report, we demonstrated that cyclosporin A, a neutral hydrophobic cyclic peptide (Log *P* = 3.64), could potentially bind to both the N- and C-terminal protein-rich domains of mucins, leading to non-covalent cross-linking that promotes the aggregation of mucin polymers [10]. The induction of mucin aggregation leads to changes in the viscoelastic properties of mucus which may provide a crucial regulatory effect on drug diffusion [20]. Therefore, we speculate that the interaction between paclitaxel and mucins may induce changes in mucin network properties, potentially influencing the cell membrane permeation of paclitaxel. Furthermore, these results support our hypothesis that interactions between mucins and drugs contribute to modulation of drug responses, and the absence of such interactions can potentially enhance drug efficacy. Therefore, our simplified assay suggests the potential to assess mucin-drug interactions, especially for those that induce mucin aggregation.

We showed that the binding affinity of drugs for polymeric mucins varied, not only among individual drugs but also among mucin subtypes. Furthermore, we have suggested that the property of drugs to induce mucin aggregation might involve a mechanism distinct from their binding affinity to mucins. We speculate that drugs with a higher binding affinity to mucins may reduce the amount of free drug on the mucosal epithelial surface, leading to a decrease in membrane permeability. Indeed, a previous study has demonstrated that the free amount of antibiotics, which included polymyxin B and daptomycin (both studied here), were decreased in the presence of porcine gastric mucin [21]. In addition, rifampicin and griseofulvin, which showed relatively high affinity for MUC2 and MUC5AC (lower than 50 μM) in this study, were reported in rats to have increased intestinal permeability after mucus was removed by dithiothreitol treatment [9]. Furthermore, 5-fluorouracil, which does not induce mucin aggregation, but has a relatively high binding affinity, exhibited alterations in dose-response behavior in A549 cells after knock-out of endogenous mucins. Our results suggested that the mucin-binding affinity of drugs is a crucial factor to consider in the pharmacokinetics and pharmacodynamics of the drugs. However, the extent of this difference does not seem to correlate with the physicochemical properties of the drug such as lipophilicity, suggesting that an undefined relationship exists in the interaction between drug and mucin. Taken together, the assessment of these interactions is expected to be a critical criterion for evaluating the efficient absorption of drugs. Further study is required to construct such an evaluation system.

To the best of our knowledge, this is the first report investigating mucin-drug interactions using purified human gel-forming mucins, MUC2, MUC5AC, and MUC5B, which are prominent in gastrointestinal (MUC2 and MUC5AC), nasal, and respiratory (MUC5B and MUC5AC) mucus [1, 8, 22]. As these mucosal surfaces are routes for drug administration, the differences in mucin subtype may be an important factor in mucin-drug interactions. The mucin-drug interactions have been explored in different approaches and different animals have been investigated as alternative mucin sources. In particular, many previous studies of porcine gastric and bovine submaxillary mucins were predominantly used to evaluate the interactions as an alternative model for human mucins [6, 16, 23, 24]. However, the composition of these commercial mucin products and the structural and biophysical characteristics of the mucins are poorly characterized and it remains unclear whether these mucins are suitable models for human mucins. Therefore, considering our findings, it is important to evaluate mucin-drug interactions using different human mucin subtypes, taking into account mucin expression patterns in the human body, and further studies would be required to elucidate such interactions.

We have demonstrated potential interactions between human mucins and various drugs by relatively simple and rapid methods. The interaction of mucins with drugs has attracted much interest in the field of drug discovery research [6, 7, 23, 25]. Going forward, it will be important to evaluate interactions with mucins in more detail for example using microscopy methods (TEM, AFM) and rheological studies, and there is a need to further develop evaluation systems that enable the rapid assessment of interactions. Our findings may provide new insights and widely applicable screening models to evaluate mucin-drug interactions. Finally, while mucin is a major determinant of the network and binding properties of mucus, and previous work has shown that the diffusion coefficients of drugs (including CsA) in purified mucin are lower than in an equivalent unstirred aqueous layer [26, 27]. We are cognizant that mucus is a complex hydrogel containing mucins alongside hundreds of non-mucin proteins and lipids. It is therefore important to recognize that these non-mucin components will likely also influence drug delivery through mucus.

In conclusion, we have shown the necessity of considering not only the potential for mucin-aggregated precipitation, but also the binding ability of compounds to mucins. However, the precise molecular mechanisms remain to be elucidated, for example identifying which domains of the mucins are important for drug binding. This systematic approach is essential for understanding how mucin-drug interactions may impact drug absorption and efficacy. Our data significantly contributes to the understanding of mucin-drug interactions, and it may ensure the efficacy and safety of pharmaceutical compounds. Furthermore, the development of a simple evaluation system for these interactions provides valuable information for drug development strategies.

## Supporting information

S1 Fig Sedimentation profiles of MUC5B in the presence of model drugs. Sedimentation profiles of MUC5B in the presence of 1 mM griseofulvin (a), 1 mM rifampicin (b), antipyrine (c), or 5-fluorouracil (d). Mucins were detected in fractions by western blotting using a specific antibody (EUMUC5B antibody) after slot blot, and the band intensities were quantified using the Odyssey Imaging system.
(TIF)

S2 Fig The fluorescence spectra and changes in maximum emission of MUC2 (10 μg/mL). Results are presented as the mean ± s.e.m. (n = 3) from 3 independent experiments.
(TIF)

S3 Fig The fluorescence spectra and changes in maximum emission of MUC5AC (10 μg/mL). Results are presented as the mean ± s.e.m. (n = 3) from 3 independent experiments.
(TIF)

S4 Fig The fluorescence spectra and changes in maximum emission of MUC5B (10 μg/mL). Results are presented as the mean ± s.e.m. (n = 3) from 3 independent experiments.
(TIF)

S5 Fig The relative secretion levels of MUC5AC and MUC5B from A549 cells. The relative secretion levels of MUC5AC (a) and MUC5B (b) from A549 cells were detected by the mucin-specific antibodies. Band intensities were quantified using the LAS-3000^®^ Imaging System. Results are presented as the mean ± s.e.m. (n = 4). *P < 0.05, **P < 0.01 compared with control condition (one-way ANOVA followed by Dunnett’s method). Grey bars = WT cells, unfilled bars = mucin KO cells.
(TIF)

S1 Raw images Full-length images of blots are presented in (A) MUC5AC for Fig 3a, (B) MUC5B for Fig 3b, and (C) β-actin for Fig 3a and 3b. Each blot was automatically imaged using a LAS-3000^®^ Imaging System (Fujifilm, Tokyo, Japan), and the protein bands shown in Fig 3a and 3b are indicated by dotted boxes. M = Molecular-weight standards (kDa).
(PDF)

S1 Table Sequences of primers used in the genome editing. (DOCX)
