# Supplementary figures and images for: Assessment of polymeric mucin–drug interactions
Source: PLoS One. 2024 Jun 27;19(6):e0306058. doi: 10.1371/journal.pone.0306058 (PMC11210812; doi:10.1371/journal.pone.0306058)

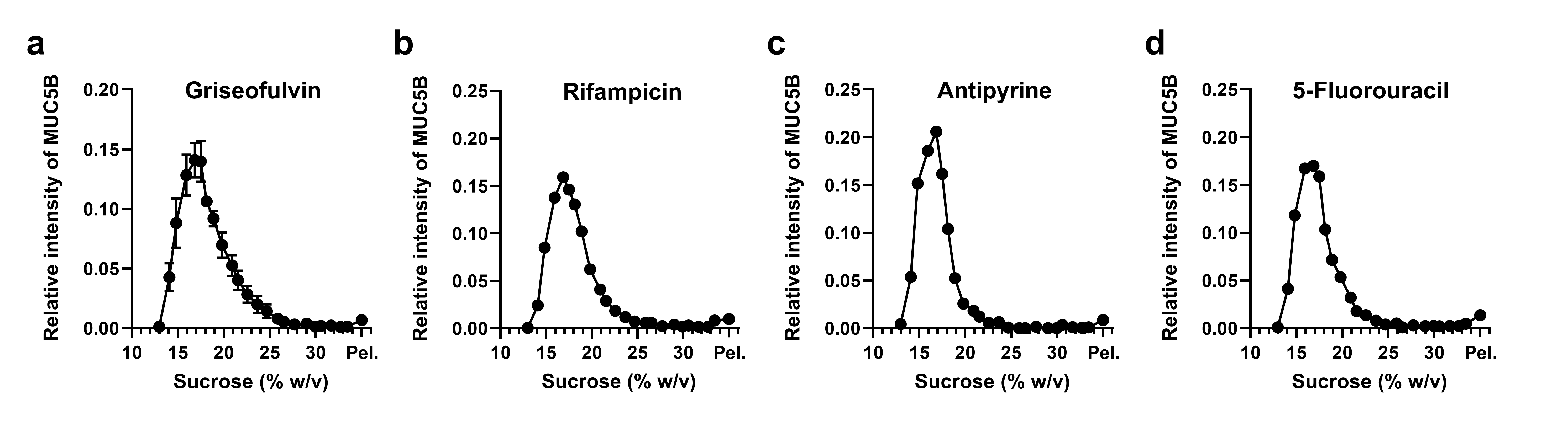

Supplement: S1 Fig — Sedimentation profiles of MUC5B in the presence of 1 mM griseofulvin (a), 1 mM rifampicin (b), antipyrine (c), or 5-fluorouracil (d). Mucins were detected in fractions by western blotting using a specific antibody (EUMUC5B antibody) after slot blot, and the band intensities were quantified using the Odyssey Imaging system. (TIF) [file pone.0306058.s001.tif]

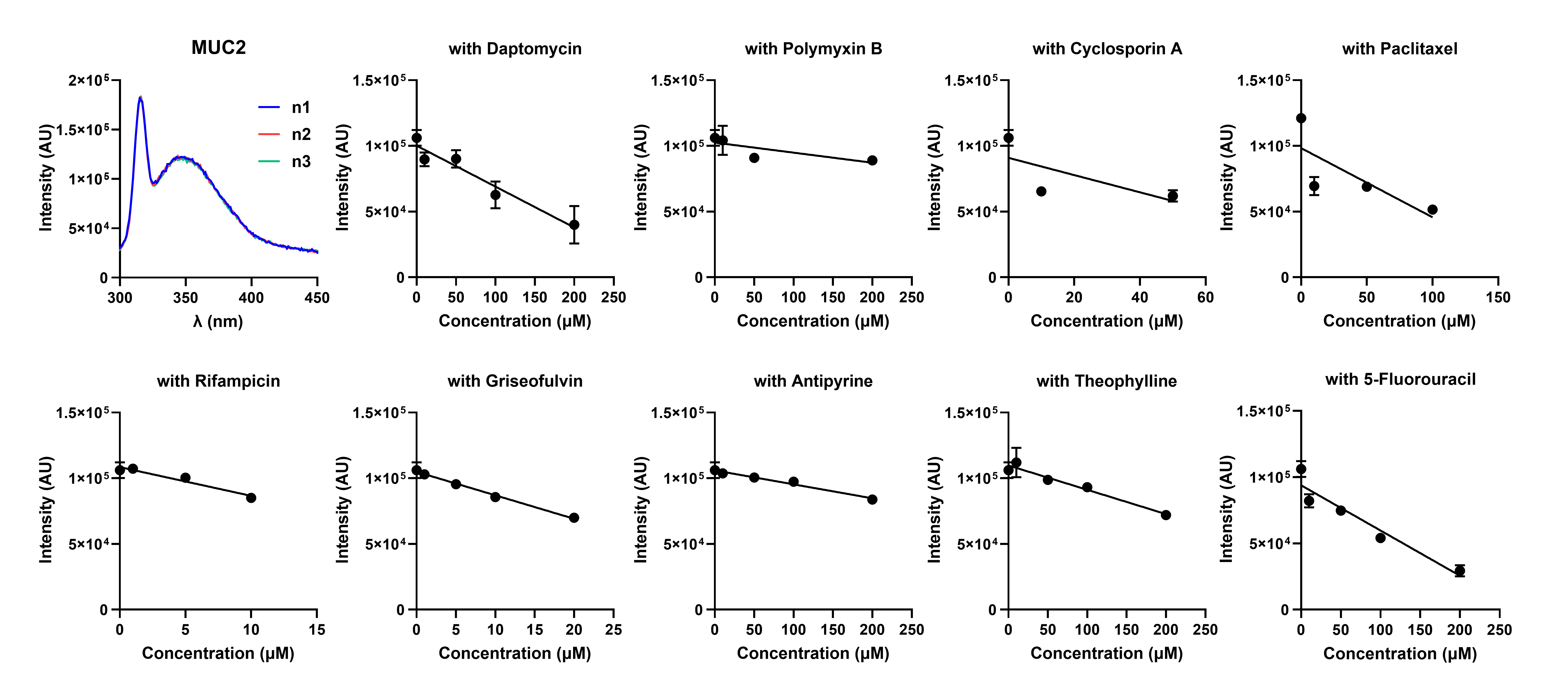

Supplement: S2 Fig — Results are presented as the mean ± s.e.m. (n = 3) from 3 independent experiments. (TIF) [file pone.0306058.s002.tif]

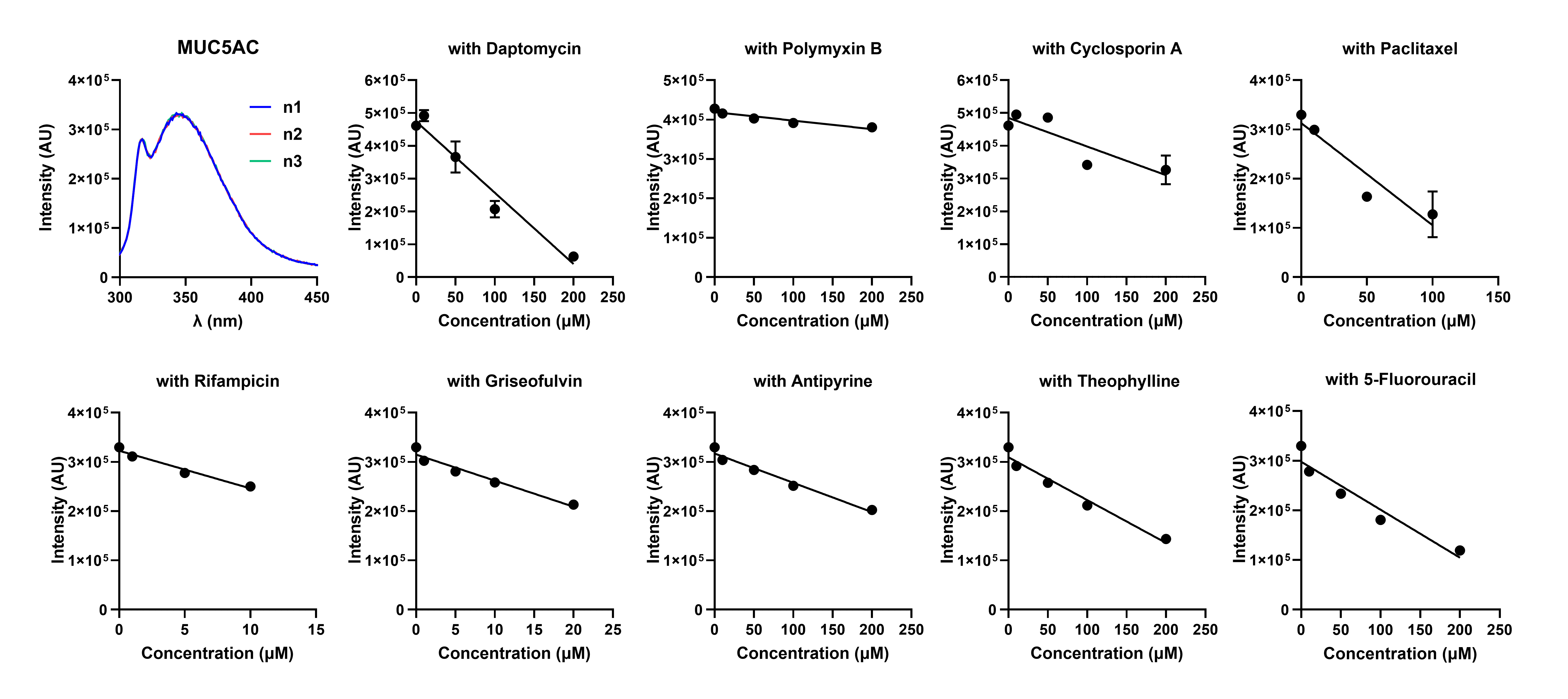

Supplement: S3 Fig — Results are presented as the mean ± s.e.m. (n = 3) from 3 independent experiments. (TIF) [file pone.0306058.s003.tif]

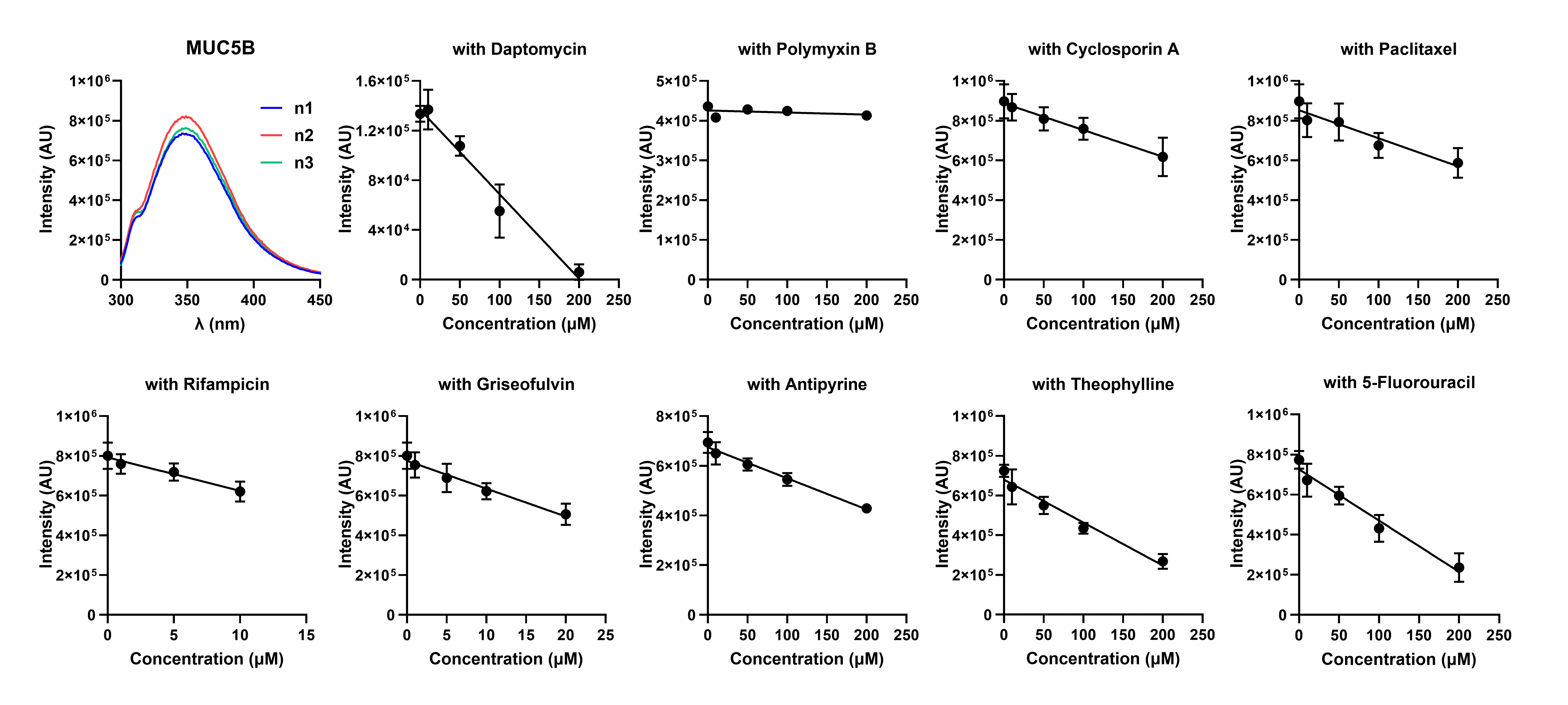

Supplement: S4 Fig — Results are presented as the mean ± s.e.m. (n = 3) from 3 independent experiments. (TIF) [file pone.0306058.s004.tif]

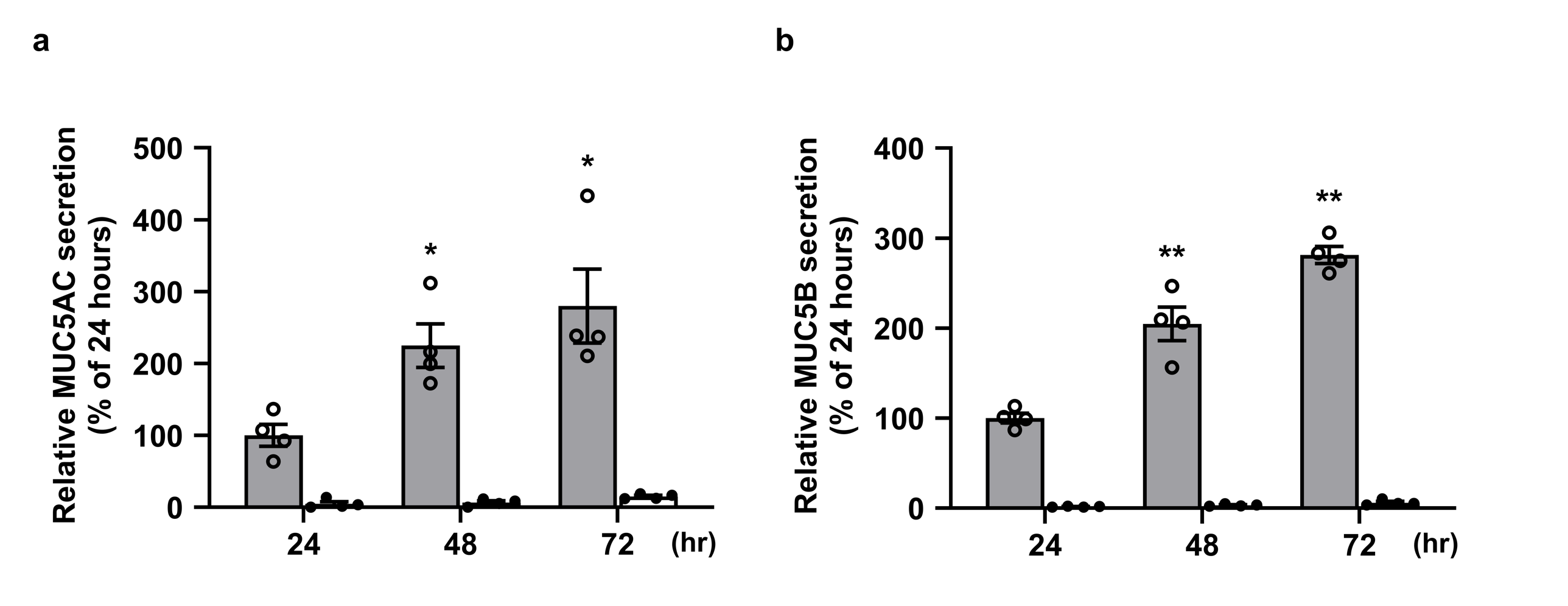

Supplement: S5 Fig — The relative secretion levels of MUC5AC (a) and MUC5B (b) from A549 cells were detected by the mucin-specific antibodies. Band intensities were quantified using the LAS-3000® Imaging System. Results are presented as the mean ± s.e.m. (n = 4). *P < 0.05, **P < 0.01 compared with control condition (one-way ANOVA followed by Dunnett’s method). Grey bars = WT cells, unfilled bars = mucin KO cells. (TIF) [file pone.0306058.s005.tif]

MUC5AC for Figure 3a

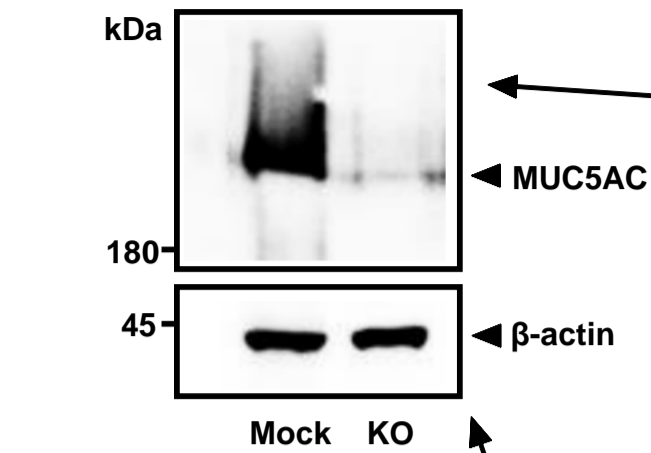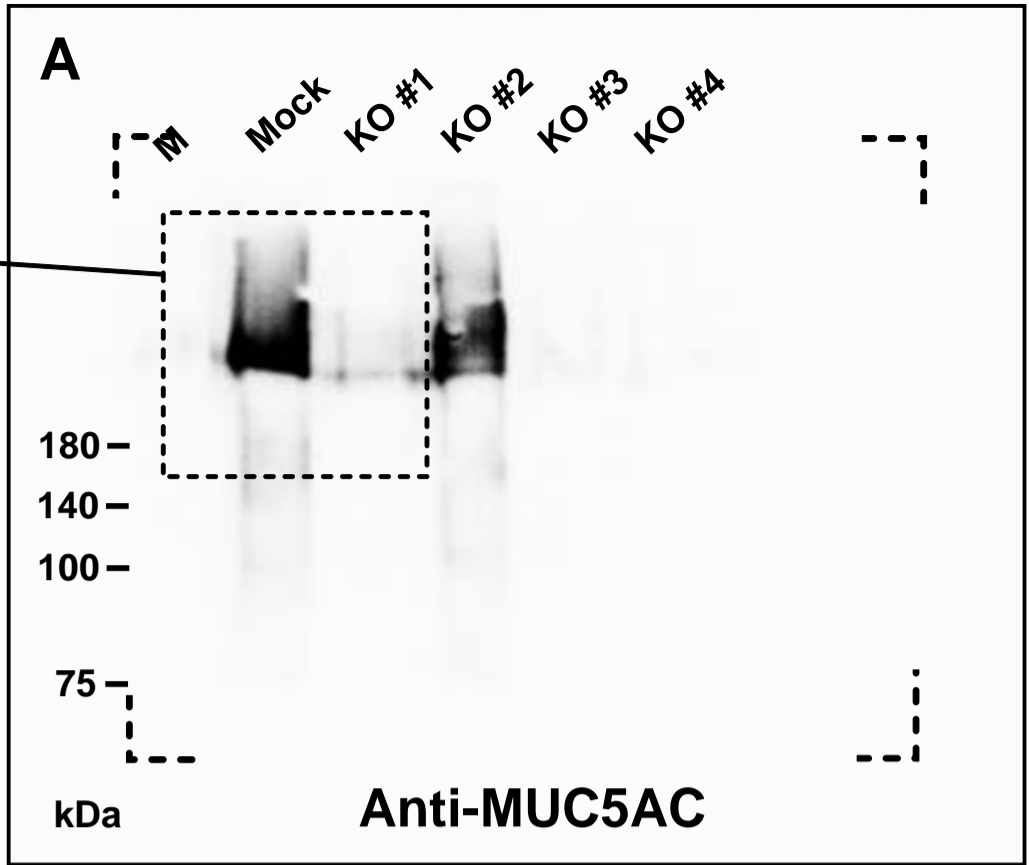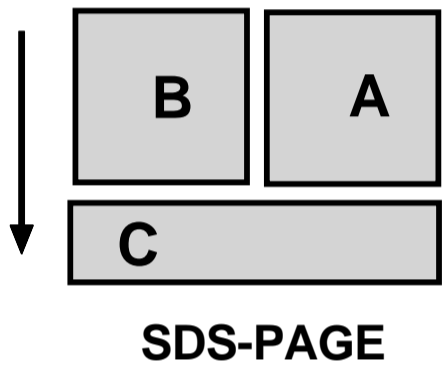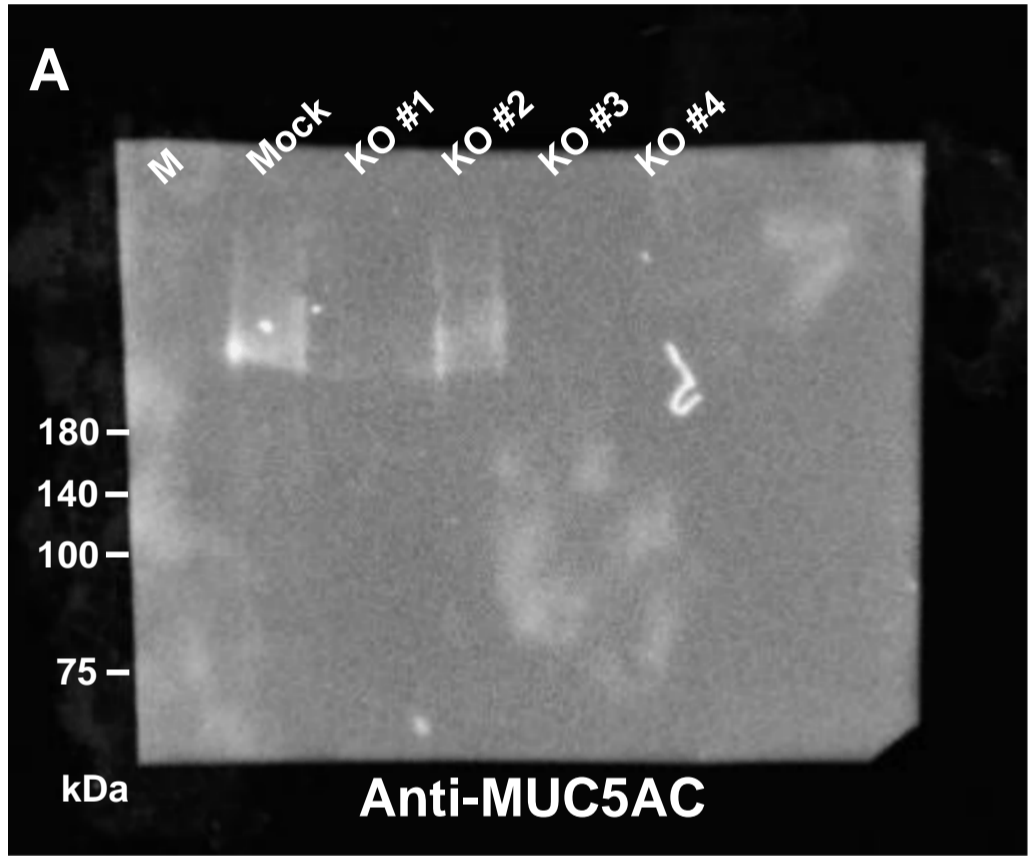

$\beta$ -actin for Figure 3a,b

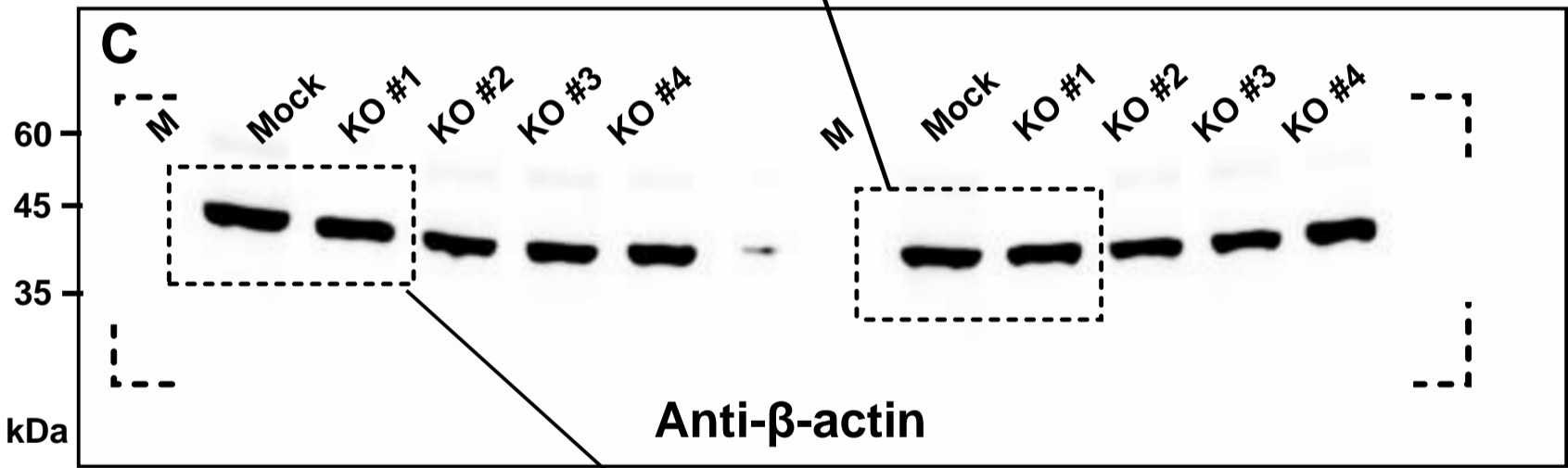

MUC5B for Figure 3b

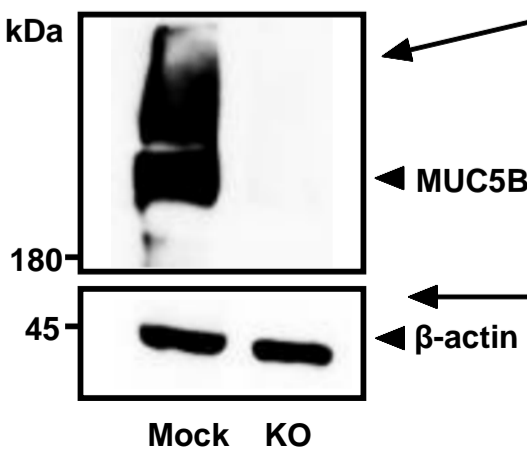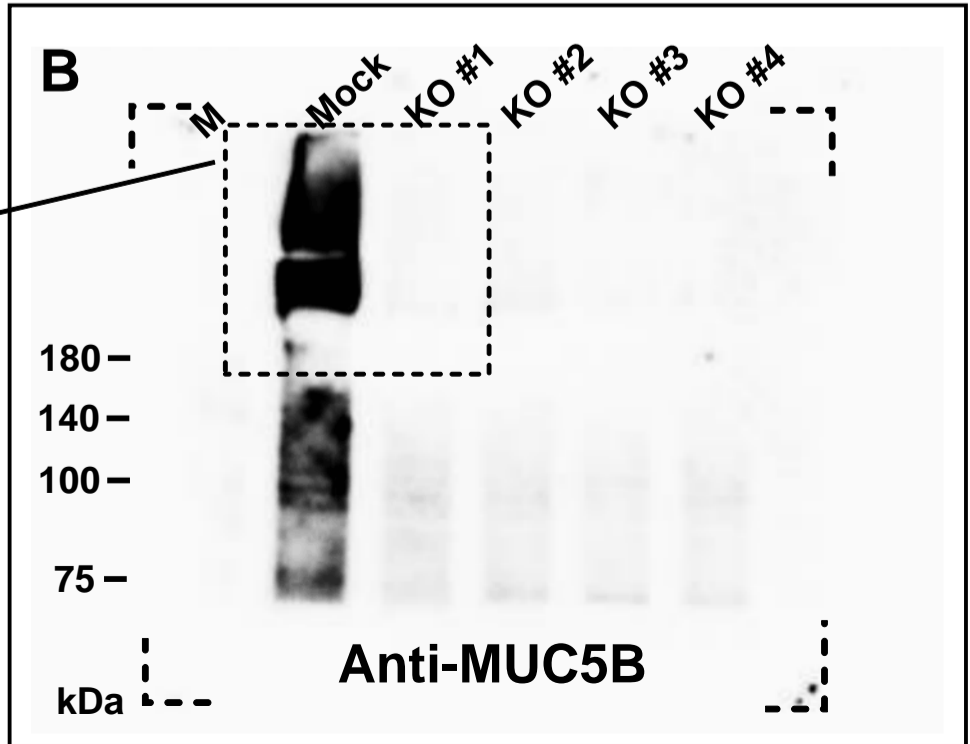

Supplement: S1 Raw images — Each blot was automatically imaged using a LAS-3000® Imaging System (Fujifilm, Tokyo, Japan), and the protein bands shown in Fig 3a and 3b are indicated by dotted boxes. M = Molecular-weight standards (kDa). (PDF) [file pone.0306058.s006.pdf]
