# Supplementary material for: Assessment of polymeric mucin–drug interactions
Source: PLoS One. 2024 Jun 27;19(6):e0306058. doi: 10.1371/journal.pone.0306058 (PMC11210812; doi:10.1371/journal.pone.0306058)
Supplement: S1 Table — (DOCX) [file pone.0306058.s007.docx]

**S1 Table. Sequences of primers used in the genome editing.**

| Gene | GenBank Accession No. | Sequences of primers and probes (5′-3′) | |
| --- | --- | --- | --- |
|  |  | **Forward Primer** | **Reverse Primer** |
| hMUC5AC_U | NM_001304359 | ATCCGCCAGGAGGTCGGCCATGGG | AAACCCCATGGCCGACCTCCTGGC |
| hMUC5AC_L | NM_001304359 | ATCCGTGGGCCCTGGCTCTCGCTC | AAACGAGCGAGAGCCAGGGCCCAC |
| hMUC5B_U | NM_002458 | ATCCGACCTGGCACCCGTCCTAGA | AAACTCTAGGACGGGTGCCAGGTC |
| hMUC5B_L | NM_002458 | ATCCGCCAGCGTCCGGCACGCGCT | AAACAGCGCGTGCCGGACGCTGGC |
